# *In silico* analysis of autoimmune diseases and genetic relationships to vaccination against infectious diseases

**DOI:** 10.1186/s12865-014-0061-0

**Published:** 2014-12-09

**Authors:** Peter B McGarvey, Baris E Suzek, James N Baraniuk, Shruti Rao, Brian Conkright, Samir Lababidi, Andrea Sutherland, Richard Forshee, Subha Madhavan

**Affiliations:** Innovation Center for Biomedical Informatics, Georgetown University Medical Center, 2115 Wisconsin Ave NW, Suite 110, Washington, DC 20007 USA; Protein Information Resource, Department of Biochemistry and Molecular & Cellular Biology, Georgetown University Medical Center, 3300 Whitehaven Street NW, Suite 1200, Washington, DC 20007 USA; Division of Rheumatology, Immunology and Allergy, Department of Medicine, Georgetown University Medical Center, 3800 Reservoir Road, NW, Washington, DC 20007 USA; Office of Biostatistics and Epidemiology, Center for Biologics Evaluation and Research, US Food and Drug Administration, 10903 New Hampshire Avenue, Silver Spring, MD 20993 USA; Department of International Health, Johns Hopkins School of Public Health, 615 North Wolfe Street, Baltimore, MD 21205 USA; Department of Computer Engineering, Muğla Sıtkı Koçman University, Muğla, Turkey

## Abstract

**Background:**

Near universal administration of vaccines mandates intense pharmacovigilance for vaccine safety and a stringently low tolerance for adverse events. Reports of autoimmune diseases (AID) following vaccination have been challenging to evaluate given the high rates of vaccination, background incidence of autoimmunity, and low incidence and variable times for onset of AID after vaccinations. In order to identify biologically plausible pathways to adverse autoimmune events of vaccine-related AID, we used a systems biology approach to create a matrix of innate and adaptive immune mechanisms active in specific diseases, responses to vaccine antigens, adjuvants, preservatives and stabilizers, for the most common vaccine-associated AID found in the Vaccine Adverse Event Reporting System.

**Results:**

This report focuses on Guillain-Barre Syndrome (GBS), Rheumatoid Arthritis (RA), Systemic Lupus Erythematosus (SLE), and Idiopathic (or immune) Thrombocytopenic Purpura (ITP). Multiple curated databases and automated text mining of PubMed literature identified 667 genes associated with RA, 448 with SLE, 49 with ITP and 73 with GBS. While all data sources provided valuable and unique gene associations, text mining using natural language processing (NLP) algorithms provided the most information but required curation to remove incorrect associations. Six genes were associated with all four AIDs. Thirty-three pathways were shared by the four AIDs. Classification of genes into twelve immune system related categories identified more “*Th17 T-cell subtype*” genes in RA than the other AIDs, and more “*Chemokine plus Receptors*” genes associated with RA than SLE. Gene networks were visualized and clustered into interconnected modules with specific gene clusters for each AID, including one in RA with ten C-X-C motif chemokines. The intersection of genes associated with GBS, GBS peptide auto-antigens, influenza A infection, and influenza vaccination created a subnetwork of genes that inferred a possible role for the MAPK signaling pathway in influenza vaccine related GBS.

**Conclusions:**

Results showing unique and common gene sets, pathways, immune system categories and functional clusters of genes in four autoimmune diseases suggest it is possible to develop molecular classifications of autoimmune and inflammatory events. Combining this information with cellular and other disease responses should greatly aid in the assessment of potential immune-mediated adverse events following vaccination.

**Electronic supplementary material:**

The online version of this article (doi:10.1186/s12865-014-0061-0) contains supplementary material, which is available to authorized users.

## Background

Vaccines are profoundly important to global health in preventing infectious diseases. However, like any medication, there are potential adverse events reported after vaccination that warrant evaluation. Adverse events reported after vaccination can be transient and common responses like fever or in rare cases, autoimmune diseases (AID) [1]. Although AIDs have been reported, to date there is no evidence to demonstrate a causal association [2]. Nonetheless, autoimmune diseases occurring after vaccination (either new onset or flairs) must be thoroughly evaluated. Biologic plausibility is one key component to reported adverse events following immunization (AEFI). Evaluation of AID as AEFI is challenging because of the complex innate and adaptive immune responses to vaccine antigens, adjuvants, excipient preservatives and stabilizers that may contribute to reactogenic responses. In addition, the genetic factors that may predispose to susceptibility to autoimmune disease are not well understood.

To contribute to the understanding of vaccinomics in the context of evaluating autoimmune AEFIs, a bioinformatics, systems biology approach was used to model the overlapping immune components involved in vaccine response and autoimmune disease. We propose that the genes and proteins that are induced or participate in vaccine immune responses, natural infections, and specific autoimmune diseases reported after vaccinations would form cross-referenced, interactive networks that could lead to hypothesis-generation for potential mechanisms and risk factors for induced autoimmune adverse events. A database was created so that the rapidly expanding universe of literature on immune mechanisms and increasingly sophisticated biological and genomic data could be searched in an expeditious manner by investigators with an interest in evaluating the safety of vaccines and other biological therapeutics. We argue that analysis of this information can significantly improve our understanding of the relevant gene networks and molecular pathways pertinent to vaccinology. Here we describe our methodology and initial results in developing curated lists of genes and vaccine components associated with autoimmune diseases and integration of this information using knowledge of biological pathways and functional processes. Our ultimate goal is to develop an online resource with genomic, immunological and molecular data to explore plausible mechanisms of autoimmune adverse event development and identification of potential risk factors for these rare events. This may contribute to hypotheses for genomic/vaccine safety studies, potential biomarkers, and improve future vaccine development.

## Results and discussion

### Selection of autoimmune diseases

The Vaccine Adverse Event Reporting System (VAERS) [3] maintained by the U.S. Center for Disease Control and Prevention (CDC) and the Food and Drug Administration (FDA) was queried for the number and type of autoimmune diseases reported following all vaccinations. The most frequently reported autoimmune diseases and the associated vaccine types are shown in Table 1. The top four AIDs reported were Guillain-Barré Syndrome (GBS), Rheumatoid Arthritis (RA), Systemic Lupus Erythematosus (SLE), and Idiopathic (or Immune) Thrombocytopenic Purpura (ITP).

**Table 1:** **Autoimmune disease reports in VAERS**

**Disease term**	**Reports in VAERS**	**Top three vaccines reported (Number of reports)**		
Guillain-Barre Syndrome (GBS)	1991	Flu (1201)	Flu H1N1 (144)	Hepatitis (127)
Rheumatoid Arthritis (RA)	403	Hepatitis (109)	Lyme (84)	Flu (47)
Systemic Lupus Erythematosus (SLE)	210	Hepatitis (90)	Human Papillomavirus (36)	Flu (23)
Idiopathic Thrombocytopenic Purpura (ITP)	180	Measles, Mumps & Rubella (64)	Varicella (46)	Flu (36)
Others (N = 39)	786			

The top four autoimmune diseases and associated vaccines Jan. 1990 - April 2012.

### Data collection and curation

The sets of genes associated with these human AIDs were obtained by using the Medical Dictionary for Regulatory Activities (MedDRA) [4] terms and variant names for each AID to screen the well-curated databases UniProt [5], OMIM [6], the Genetic Association Database (GAD) [7], KEGG Pathways [8] and Immune Epitope Database (IEDB) [9]. The biomedical literature contained many more AID associated genes than the curated data sources. Additional genes were found using the Pathway Studio 9 (PS9) software package [7] and its internal ResNet 9 Mammalian database to text-mine PubMed. The PS9 gene associations were manually screened for validity before adding them to gene lists from the curated databases. Approximately 20% of the gene associations found via text mining were rejected after review, 621 associations remain. All genes from all sources were compared in pairwise fashion for the four AIDs (Table 2). Each data source provided important and unique gene associations and the overlap between them was low. The data indicate that the use of multiple databases coupled with active text mining of the literature were essential to generate a well-supported and relatively complete list of gene - disease associations. Automated text mining using NLP algorithms provided the most associations but required some manual effort to confirm the associations.

**Table 2:** **Pairwise comparison of gene to autoimmune disease data sources**

	**GAD**	**IEDB**	**KEGG**	**OMIM**	**PS**	**UniProt**	**Unique to source**
**GAD**	**174**	**8**	**32**	**17**	**95**	**28**	**71**
**IEDB**		**156**	**13**	**3**	**45**	**23**	**93**
**KEGG**			**172**	**4**	**67**	**21**	**89**
**OMIM**				**40**	**19**	**18**	**17**
**PS + Curation**					**621**	**50**	**434**
**UniProt**						**112**	**38**

Common and unique genes associated with one or more of four AIDs (RA, SLE, ITP, GBS). Sources are UniProt, OMIM, Genetic Association Database (GAD) KEGG Pathways, IEDB and the curated list originally derived from Pathway Studio (PS).

Our collection contains 667 genes associated with RA, 448 with SLE, 49 with ITP and 73 with GBS. Many genes were associated with multiple AIDs. The list of genes associated with each AID including additional functional information on each gene is provided in Additional file 1. The literature sources for gene to disease association are contained in Additional file 2 and consist of a database reference and/or a PubMed identifier for a publication that claims an association. This gene set represents the largest, most complete, high quality source of genes and protein products associated with these four AIDs. DisGeNet [10] provides a similar quality resource with computationally mapped and classified genes associated with many diseases from multiple data resources including text mining. A comparison between our gene lists and those provided by DisGeNet for the four AIDs showed substantial overlap but with more genes identified in our more focused resource. This is likely due to some differences in the original data sources used and differences in methods used for mining the data sources we have in common. In addition our manual screening of associations derived from text mining, eliminated some erroneous associations.

Gene interactions with vaccine ingredients were obtained from the curated Comparative Toxicogenomics Database (CTD) [11] and PubChem [12]. The CTD list was filtered to include direct ingredient to gene interactions with indirect interactions, those with intermediate genes or compounds, removed. The filtered list contained 64 interactions between 6 ingredients and 46 genes (Additional file 3). However, these sources are incomplete from an adjuvant perspective. Alum is the most commonly used adjuvant, but this term is generic and encompasses several aluminum salts such as aluminum hydroxide, phosphoaluminum sulfate, and aluminum sulfate. The toxicological databases do not reflect this diversity since entries were found only for aluminum sulfate and the general term aluminum. Few studies of the effects of these adjuvants on human leukocyte or other transcriptomes were available.

### Functional analysis and classification

Functional annotations for each gene/protein were collected from UniProt [13], Reactome BioMart [14] Protein Information Resource [15] and Gene Ontology (GO) [16]. Annotations included alternate gene names, protein names, function (if known), involvement in other diseases and pathways. Immune system gene classifications were obtained from the ImmPort website (http://immport.niad.nih.gov). Annotations are included in Additional file 1.

**Figure 1:**
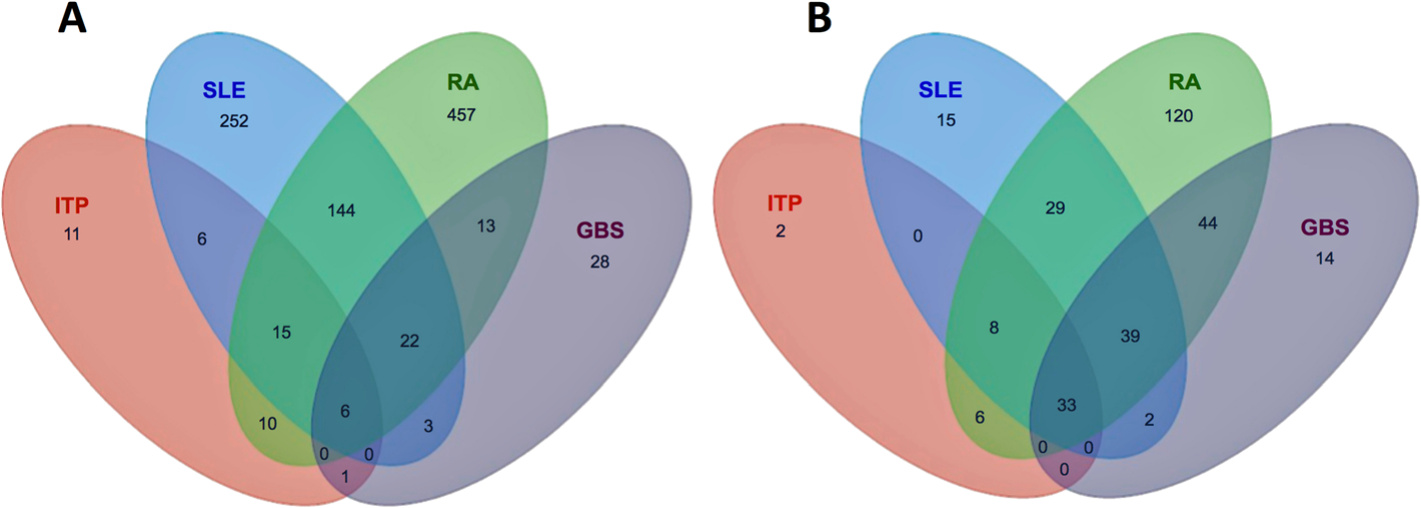
**Venn diagrams comparing genes and pathways associated with autoimmune diseases. A** – Numbers of common and unique genes. **B**- Numbers of common and unique pathways. Details on genes and pathways in Additional files 1 and 2. Statistical analysis details in Additional file 9: Table S9.

Functional networks were created for each AID using Cytoscape [17] and the ReactomeFI plug-in [18,19]. The ReactomeFI tool was used to perform pathway and GO enrichment analysis. The numbers of unique and common genes and pathways observed for each AID were compared (Figure 1). Pathway analysis of the four AIDs identified pathways from four sources KEGG, BioCarta (http://www.biocarta.com), the Pathway Interaction Database (PID) [20] and Reactome [21]. All pathways identified for each AID (312 total) and the genes that mapped to each pathway are included in Additional file 4. Detailed Pathway and GO analysis results for each network are contained in the Additional files 5, 6, 7 and 8 for each network. Overlaps in genes and pathways associated with four diseases were shown to be significant using multiple methods.

Only six genes (TGFB1, IFNG, CD4, FCGR3A, FCGR2A, HLA-DRB1) and thirty-three pathways were common to all AIDs. Not surprisingly all six common genes are well known to participate in many immune response and disease pathways. CD4 and INFG implicate TH1 lymphocytes. TGFB1 suggests roles for T-regulatory cells. The two IgG FC receptors suggest antibody mediated immunopathology or immunomodulation. HLA-DRB1 suggests antigen processing for presentation to T-cells. Twenty-two genes were common to RA, SLE and GBS, and RA, SLE and ITP shared fifteen genes. Two hundred and twenty genes were associated with two or more AIDs. This list is likely to expand as more studies are reported.

The thirty-three pathways shared by the four AIDs were likely due to inclusion of well-characterized signaling proteins that have been implicated in many pathways and functional processes. Fourteen of the thirty-three were KEGG disease pathways. Pathways related to natural influenza and other infections were included to cross-reference mechanisms potentially related to disease-induced AIDs. The pathway list may be somewhat redundant since the four individual pathway databases overlap and have differences in classifying the boundaries of a pathway. It is likely some interactions appear in multiple pathways.

The complete gene set was classified into twelve immune system related categories and sub-categories plus two categories for immune diseases and infectious diseases (Table 3). This mechanistic approach represents a first approximation for a molecular classification of AIDs. Categories for which disease-associated genes are significantly enriched are highlighted in light gray. The large number of genes and pathways associated with RA and SLE compared to ITP and GBS probably represents a research bias given the extensive study of these diseases and identification of the probable contributions of the most important pathophysiologically relevant systems. RA and SLE had similar patterns of classification for most genes and pathways. The major difference between them was in the category of “*Cytokines and Cytokine Receptors*” where RA had more genes in the *“Chemokine and Receptors”* subcategory than SLE. This distinction has been noted previously in genome wide association studies in autoimmune diseases [22]. Also of interest is the relatively high percentage of GBS associated genes in *“Infectious Disease Pathways”*. Again consistent with the reports in the literature that GBS often follows various bacterial and viral infections [23].

**Table 3:** **Disease associated genes in different categories and pathways**

	**Rheumatoid arthritis**	**Systemic lupus erythematosus**	**Immune thrombocytopenic purpura**	**Guillain-barre syndrome**
**Number of Genes Associated >>**	667	448	49	73
**v Categories v**				
Antigen Processing & Presentation	**6.30%**	**9.15%**	**14.29%**	**8.22%**
Antimicrobials	**19.64%**	**17.19%**	**28.57%**	**39.73%**
BCR Signaling Pathway	**2.85%**	**2.23%**	**6.12%**	**4.11%**
Cytokines + Receptors	**28.49%**	**18.97%**	**30.61%**	**24.66%**
> Chemokines + Receptors	**7.05%**	**3.79%**	4.08%	**5.48%**
> Interferons + Receptors	0.30%	0.67%	4.08%	**4.11%**
> Interleukins + Receptors	**5.55%**	**4.02%**	**8.16%**	5.48%
> TGF-b Family Members + Receptors	**1.20%**	0.45%	2.04%	**2.74%**
> TNF Family Members + Receptors	**3.00%**	**2.90%**	**4.08%**	1.37%
Natural Killer Cell Cytotoxicity	**6.45%**	**6.47%**	**12.24%**	**9.59%**
TCR Signaling Pathway	**4.50%**	**4.46%**	**10.20%**	**10.96%**
Other Immune-Related	**28.94%**	**29.46%**	34.69%	19.18%
Immune Disease Pathways	**19.34%**	**31.47%**	**38.78%**	**28.77%**
Infectious Disease Pathways	**28.79%**	**29.46%**	**34.69%**	**45.21%**
Unclassified	**16.49%**	**19.20%**	**8.16%**	**17.81%**

Chemokine’s and their receptors were grouped together. Percentages do not sum to 100% as categories can overlap. Bold numbers indicate significant results using Fishers exact test with Bonferroni correction. Data is in Additional files 1 and 9.

T-cell subsets that may have been specifically involved in each AID were investigated based on consensus information about the most discriminating cell surface antigens, transcription factors, secreted effector molecules, and distinct functional roles of subtypes of T lymphocytes and other immune cells (www.nature.com/nri/posters/tcellsubsets/index.html). RA was associated with a larger number of Th17 related genes than the other AIDs (Table 4). This was consistent with experimental observations of Th17 cells in human synovial fluid and theories of RA pathogenesis [24,25]. The large number of genes associated with T follicular helper (TFH) cells supported clinical observations in RA [26] and SLE [27] regarding this cell’s importance in autoimmune diseases [28].

**Table 4:** **Genes associated with T-cell types**

	**RA**	**SLE**	**ITP**	**GBS**	**All four AIDs**
**Cytotoxic T cell (n = 13)**	**7**	**8**	**1**	**1**	**7**
**Exhausted T cell (n = 7)**	**4**	**5**	**0**	**0**	**6**
**Anergic T cell (n = 11)**	**4**	**2**	**0**	**0**	**5**
**TR1 cell (n = 9)**	**4**	**4**	**1**	**2**	**5**
**Natural TReg cell (n = 15)**	**8**	**8**	**2**	**3**	**9**
**Inducible TReg cell (n = 17)**	**8**	**8**	**2**	**3**	**9**
**NKT cell (n = 10)**	**6**	**5**	**2**	**1**	**10**
**CD8αα** **T cell (n = 10)**	**7**	**7**	**1**	**1**	**8**
**CD4+ αβ** **T cell (n = 10)**	**3**	**4**	**1**	**1**	**4**
**CD8+ αβ** **T cell (n = 9)**	**3**	**4**	**0**	**0**	**3**
**TH1 cell (n = 14)**	**8**	**9**	**3**	**4**	**10**
**TH 2 cell (n = 19)**	**8**	**8**	**2**	**2**	**10**
**TH 9 cell (n = 8)**	**3**	**4**	**1**	**2**	**4**
**TH 17 cell (n = 20)**	**11**	**5**	**1**	**1**	**12**
**TH 22 cell (n = 8)**	**3**	**3**	**1**	**1**	**4**
**TFH cell (n = 16)**	**9**	**10**	**2**	**3**	**13**
**Central memory T cell (n = 18)**	**7**	**10**	**3**	**1**	**10**
**Effector memory T cell (n = 8)**	**2**	**5**	**0**	**0**	**5**
**γδ T cell (n = 10)**	**5**	**4**	**1**	**1**	**7**

Genes for the surface phenotype, transcription factors, secreted effector molecules and other functions of different classes of T-cells were identified for each AID. The number in parentheses next to the cell type represents the total possible for each cell type. The number in each cell represents the number present in the AID gene set.

Although the smaller numbers of genes and pathways for ITP and GBS represent the best available sample from the current literature, they were unlikely to present the complete mechanistic schema. GBS had a much larger proportion of genes in the antimicrobial and infectious disease categories (Table 3), which were consistent with the association of GBS and Campylobacter, influenza, and other bacterial and viral infections [29].

### Gene networks, data integration and hypothesis building

These gene associations were derived from experimental data about mechanisms of autoimmune diseases, infectious diseases and vaccination biology. Analysis of them provides insights into molecular mechanisms of autoimmune disease and can assist in the development of new hypotheses that promote vaccine safety. Gene interaction networks were built for each AID and clustered into interconnected modules. The network data and Cytoscape files for each are included in the supplemental material.

**Figure 2:**
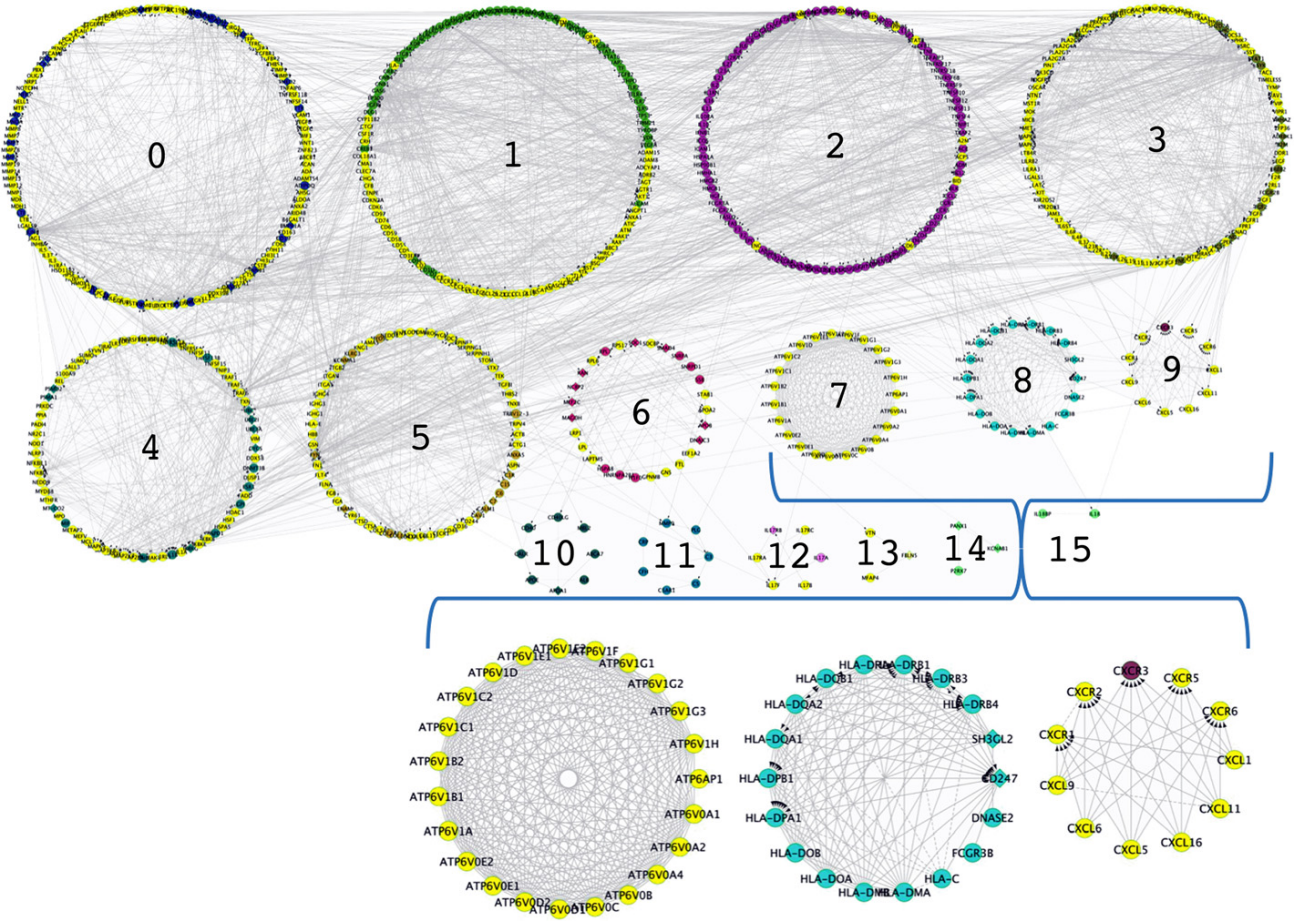
**Functional interaction network of Rheumatoid Arthritis associated genes.** The network was created and visualized using Cytoscape and the ReactomeFI plug-in. The genes were clustered into 16 interconnected modules using spectral partition network clustering [66] as implemented by the ReactomeFI plug-in. Gene nodes in yellow are genes associated with RA and none of the other four AIDs in this study. Modules 7, 8, 9 are enlarged. Additional images and full details of all genes in each module are provided in Additional file 5: Table S5.

In the functional interaction network for RA (Figure 2), the genes highlighted in yellow were associated with RA only and not the other AIDs. The network clustered into 16 modules composed of RA-selective genes as well as genes shared by multiple AIDs. For example, the genes in modules 7 and 9 were generally limited to RA. Identification of functional gene clusters unique for a particular AID and those common for multiple AIDs can inform future studies on potential therapeutics and vaccines. Module 7 is entirely composed of V-type proton ATPase subunits and isoforms. These protein pumps acidify phagolysozomes to promote microbial killing and other cellular processes. A subset of these genes and proteins were involved in ostoclast induced bone readsorption as occurs under inflammatory conditions in RA [30,31]. Some variants of these proteins may be involved in T-cell activation [32,33]. Module 9 was composed entirely of C-X-C motif chemokines and chemokine receptors. According to UniProt Knowledgebase (UniProtKB) annotation, most were powerful neutrophil chemotactic factors. CXCR1 and CXCR2 are receptors for IL8 and potent neutrophil chemoattractants. CXCL9 and CXCL11 are chemotactic factors for T-cells. CXCR5 is a chemotactic factor for B-cells. Three C-X-C motif chemokines or receptors from module 2 were associated SLE and RA. In general, more chemokines and chemokine receptors were associated with RA than SLE (Table 3). Genes in modules 2 and 8 were associated with multiple AIDs. Module 2 had one hundred genes, but only ten were only associated with RA only. Four genes were associated with RA, SLE, GBS and ITP. The largest functional group of genes in module 2 were cytokines and cytokine receptors including fourteen members of the Tumor Necrosis Factor superfamily, thirteen interleukins, eight chemokines and three macrophage stimulating factors. Module 8 had fourteen major histocompatability (MHC) class II antigen presentation genes plus genes for IgA production pathways. Data on this network including all genes, the clusters, pathways and GO analysis for each individual module are included in Additional file 5: Table S5.

As another example, Guillain-Barré Syndrome (GBS) is an acute polyneuropathy with demyelination of the peripheral nervous system. Its causes are not fully known, but about one third of cases are preceded by *Campylobacter jejuni,* cytomegalovirus, influenza, and other bacterial and viral infections [22]. The influenza virus prepared for the 1976–77 swine flu pandemic [34,35] led to an excess of GBS cases within 42 days of immunizations. This created a legacy of warnings for influenza vaccine – related to GBS. Retrospective studies have not demonstrated an increased risk for GBS [36]. Prospective tracking and meta-analysis of the 2009 Influenza A (H1N1) monovalent inactivated vaccine in the U.S. concluded there were 1.6 excess cases of GBS per 1 million persons vaccinated [37]. This is similar to rates after natural influenza infection. However, a global consortium analyzed Influenza A (H1N1) 2009 monovalent vaccination and reported measurable risk of GBS [38]. Investigations of persons who develop GBS after influenza infections or immunization may identify genetic risks factors related to GBS pathophysiology.

**Figure 3:**
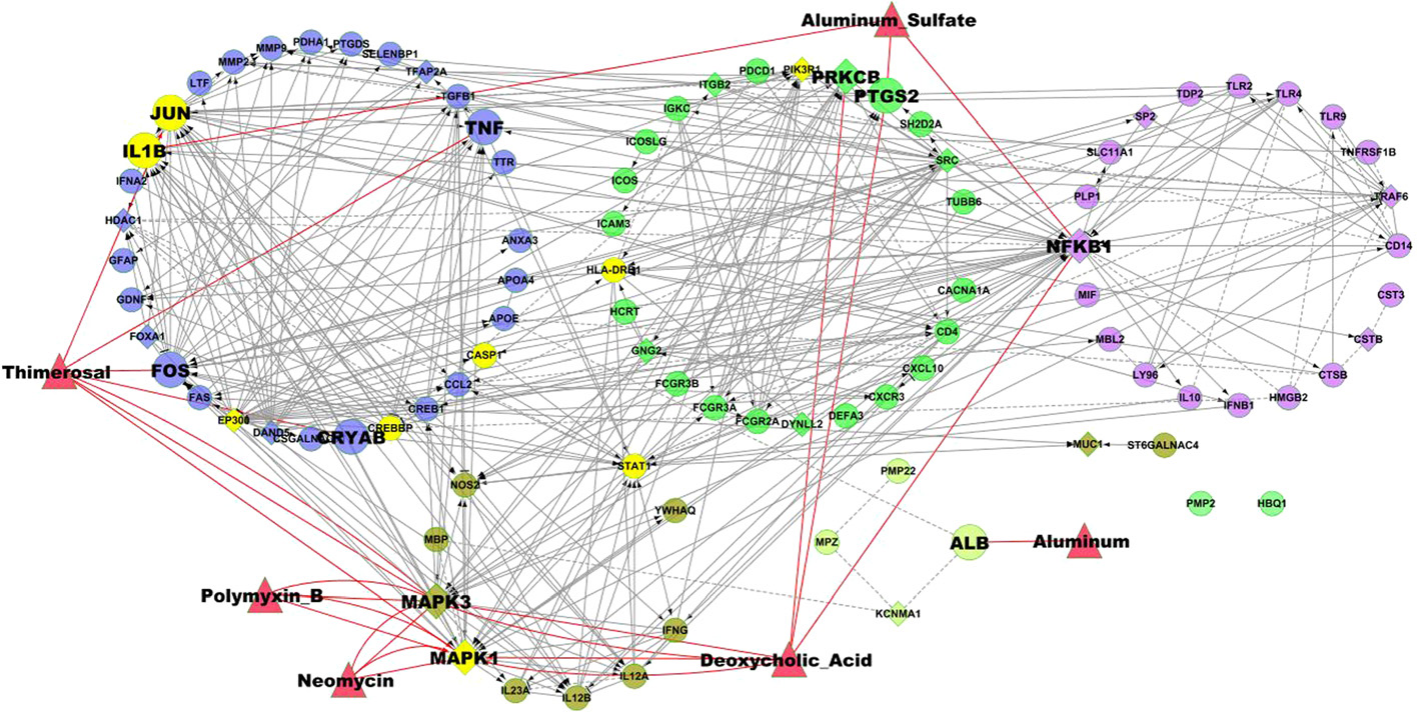
**Functional interaction network of Guillain-Barre Syndrome associated genes and vaccine ingredients.** Genes associated with GBS are represented by circles. “Linker” genes added interconnect the network are represented as diamonds. Red triangles represent vaccine ingredients that interact with genes in the network. Genes highlighted in yellow are present in both the KEGG Influenza A pathway and were significantly up or down regulated following influenza vaccination [39]. The circles of similar color are “modules” from clustering based on network topology. Full details of all genes in each module are provided in Additional file 6: Table S6.

**Figure 4:**
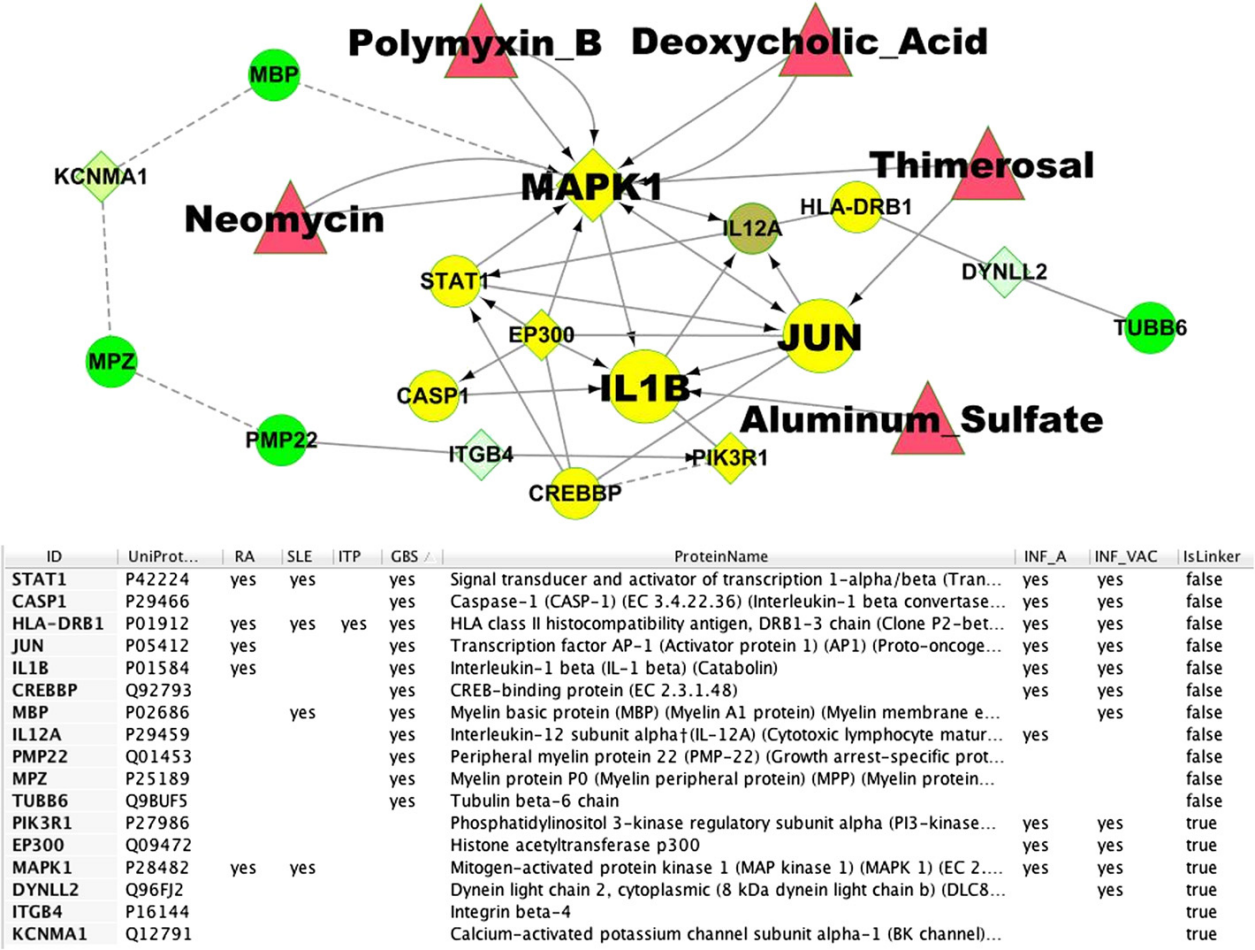
**Subnetwork of genes associated with Guillain-Barre Syndrome, influenza A infection, influenza vaccination and GBS auto-antigens.** The subnetwork was created from the genes highlighted in Figure 3 (shown in yellow) plus vaccine ingredients (shown in red triangles) and peptide epitopes related to GBS from IEDB (shown in green) A minimal set of four linker genes from Figure 3 (diamond shaped nodes) plus IL2A were included to connect all nodes in the subnetwork.

A GBS gene network was created and clustered into interconnected modules. Interactions between vaccine ingredients and genes in the network were added. In addition we identified all genes in the network that were in the annotated KEGG Influenza A infection pathway and also all genes significantly up and/or down regulated following influenza vaccination [39]. The network is shown in Figure 3. Nine genes in the network were present in all three data sets (*GBS network, Influenza pathway* and response to vaccination) and highlighted in yellow. To investigate further relationships between these genes we created a subnetwork consisting of the nine genes plus the four genes identified by IEDB as peptide epitopes in GBS (Figure 4). The subnetwork contained sixteen genes, eleven were associated with GBS, ten were part of the KEGG “Influenza A” pathway and eleven showed significant expression changes after vaccination. The other five genes not associated with GBS were added as linker genes to connect portions of the network. Though not associated directly with GBS, three linker genes were associated with influenza infection and vaccination and one with influenza vaccination only. Pathway analysis found that 5 or more of the genes were included in five non-disease specific pathways: “*IFN-gamma pathway*” (PID); “*toll-like receptor signaling pathway*” (KEGG); “*signaling by interleukins*” (Reactome); “*glucocorticoid receptor regulatory network*” (PID); and “*JAK-STAT signaling pathway*” (KEGG).

Five genes were uniquely associated with GBS and not with RA, SLE or ITP including MPZ (Myelin protein P0), TUBB6 (Tubulin beta-6 chain), PMP22 (Peripheral myelin protein 22), which are auto-antigens, plus CASP1 (Caspase 1), CREBBP (CREB-binding protein), and IL12A (Interleukin-12 subunit alpha). Links to UniProtKB, KEGG pathways, and annotated literature collected in this study suggested mechanistic implications. CREBBP and EP300 (Histone acetyltransferase p300) acetylate histone and non-histone proteins for transcriptional regulation of antiviral interferons [40-45]. JUN is proposed to help promote IL1 and IL12 expression in influenza A. Microarray studies of cultured lymphocytes show that the vaccine ingredient thimerosal increased expression of JUN [46].

PIK3R1 and CASP1 interact with the influenza A NS1 protein. PIK3R1 (phosphatidylinositol 3-kinase regulatory subunit alpha) interacts with multiple proteins and signaling pathways. PIK3R1 is activated by double stranded RNA docked on NS1 (dsRNA-loaded NS1) and may promote viral replication by inhibiting premature apoptosis and promoting viral protein expression and nucleocapsid export [47-49]. PIK3R1 also has an upstream role in promoting the production of interferons via CREBBP and EP300. CASP1 is a component of the inflammasome formed in response to viral infection and cleaves the precursor forms of IL1B and IL18 into mature forms that are released from macrophages [50]. NS1 may inhibit CASP1 and the production of IL18 [51]. IL1B was also in the subnetwork, and is involved in stimulation of ”*thymocyte proliferation*”, “B*-cell maturation and proliferation*”, and “*fibroblast growth factor activity*”. The IL1B precursor mRNA was significantly up-regulated by aluminum sulfate in neural cell cultures [52]. STAT1 (signal transducer and activator of transcription 1-alpha/beta) functions as a signal transducer and transcription activator that mediates responses to interferons, cytokines and growth factors via the Jak/STAT pathway. Its role in influenza A may be to promote expression of antiviral proteins and initiate expression of HLA class II genes like HLA-DRB1 that were also present in the subnetwork.

One of the linker genes added to the network was MAPK1 (mitogen-activated protein kinase 1), a multifunctional serine/threonine kinase that is an essential component of the MAP kinase signal transduction pathway along with STAT1 and JUN. Though not directly associated with GBS in our data set, MAPK1 was an extensively connected vertex that contacted almost all of the genes in this network. MAPK1 was associated with RA, SLE, influenza A infection, and was up-regulated following trivalent inactivated Influenza vaccine (TIV) and live-attenuated influenza vaccine (LAIV) administration [39]. Gene expression studies in peripheral blood leukocytes from GBS patients found the MAPK signaling pathway to be one of the most significantly up-regulated pathways [53]. In addition, the vaccine ingredients polymyxin B, neomycin, and deoxycholic acid increased MAPK1 phosphorylation and activity [54-56], while thimerosal may decrease its activity [57]. In influenza A, MAPK appears to promote viral protein expression and nucleocapsid export. PIK3R1 may have similar functions but the exact mechanisms remain unclear.

On the periphery of the subnetwork in Figure 4 with fewer connections to other genes in the network are four genes that IEDB identified with peptide epitopes in GBS: MBP (myelin basic protein), MPZ (myelin protein P0), PMP22 peripheral myelin protein 22), and TUBB6 (tubulin B6). MBP, MPZ and PMP22 were predicted to be connected to each other and eventually to MAPK1 (indicated by dotted lines) based on the shared GO biological process annotation “*synaptic transmission*” (in UniProtKB), and membership in an “*axon guidance*” canonical pathway in Reactome. MBP, MPZ and PMP22 received this GO annotation based on maintenance of myelin sheath integrity and mutations that cause demyelination [58-60]. MAPK signaling has been shown to be involved in the demyelination process and in Schwann cell differentiation [61,62] although full details of all the molecular mechanisms remain to be determined. TUBB6 is connected via an interaction with DYNLL2 (Dynein light chain 2) in transporting antigen loaded MHCII molecules to the cell surface.

## Conclusions

Improved understanding of the systems biology related to adverse events occurring after vaccines and medications in general is critical to enhancing the efforts to evaluate safety. One first step is to identify all the genes and molecular processes and pathways involved. The data presented analyzed genes involved in four autoimmune diseases commonly reported as following viral infections and also reported following vaccination against the virus. Our analysis has identified common and unique genes and pathways for each AID. Classification of genes into immune system categories identified more “*Chemokine plus Receptors*” genes associated with RA than SLE. RA also had more genes associated with the “*Th17 T-cell subtype*” than the other AIDs. These results suggest it is possible, with additional data and effort to develop molecular classifications of autoimmune and other inflammatory events. Combining this information with cellular and other disease responses [1] should greatly aid in the assessment of potential immune-mediated adverse events following vaccination.

A benefit of having a reliable curated list of gene associations is that it facilitates integration and analysis with other data resources and experimental data from the literature, to develop hypotheses, enhance understanding of the systems biology of vaccines and vaccine preventable diseases. Some limitations to this approach are that the body of knowledge in the literature is incomplete, imperfect and biased toward specific diseases that affect more individuals and receive corresponding increases in research and funding.

Network analysis of AIDs demonstrated integration and analysis from outside resources by using the gene lists to build functional gene interaction networks using data from multiple databases collected in the ReactomeFI tool. The analysis for RA allowed identification of functional gene clusters unique for RA and clusters common for multiple AIDs. Analysis of the GBS network included data from KEGG’s Influenza A infection pathway and experimental data from a systems biology study of influenza vaccination. This helped to define a subnetwork of genes and pathways involved in all three processes *Influenza infection, Guillain-Barré Syndrome and Influenza vaccination* and inferred a possible role for the MAPK signaling pathway in influenza vaccine – related GBS.

Systems, methods and tools to collect organize and integrate the increasing volumes of data are essential for medical researchers and regulatory agencies to evaluate molecular data and develop testable hypotheses related to vaccine safety and efficacy. Literature mining together with rigorous network modeling and statistical approaches can help improve vaccine safety monitoring and evaluation. We hope this data will inform experimental studies on the relationships between these diseases and vaccination, assist in the analysis of new experimental data, new *in silico* models of vaccine related adverse events, and in the development of novel therapeutic strategies. Such models will help enable rapid classification of immune-mediated diseases. Our results and observations are based on what is available in published literature and genetic databases to date. We expect the list of gene associations to grow, especially for ITP and GBS. As more studies are conducted, a more complete picture of the genetic map associated with these diseases will emerge. All comparisons between AIDs should be viewed with these caveats in mind. Importantly, any of the suggestions we may infer in our analysis here still need to be further evaluated in independent studies (e.g., using prospective study designs) to confirm any of the results.

## Methods

### Data collection and curation

To define an initial set of autoimmune diseases we downloaded and queried the national VAERS [3] reports submitted between Jan 1990 and April, 2012. SAS software was used to query the dataset. Lists of vaccines, their manufacturers with brand names, associated adverse events and numbers of cases reported are collected. Using the MedDRA [4] thesaurus from BioPortal [63] all the adverse events that fall under “Autoimmune disorders” term were filtered. Four autoimmune diseases Rheumatoid arthritis (RA), Systemic Lupus Erythematosus (SLE), Guillain-Barre Syndrome (GBS) and Immune Thrombocytopenic Purpura (ITP) were chosen for further study to collect genes associated with each disease. The goal was to generate a high quality list of human genes associated with the four AIDs. The criteria for inclusion was broad in that the gene association could be of any type such as significant changes in gene or protein expression, GWAS association, sequence variations associated with the disease and others. The criteria were strict in that our sources must be either from a well-respected human curated database and/or traceable to published literature. As much as possible evidence was restricted to be from human studies not mouse model systems, though often mouse and human studies are presented in the same publication. Only protein-encoding genes were included and most associations with a genomic locus without a known gene were excluded. The one exception to this was SLE where several well-studied loci associated with inherited SLE from OMIM were included. Additional associations to non-protein encoding loci may be included in the future. Genes with associations from high-throughput analysis only, for example genes up-regulated in a relevant microarray experiments, were not included in the associated set unless the published evidence contained additional corroborating data for an association. Initial sources included UniProt [5], OMIM [6], the Genetic Association Database (GAD) [7], and KEGG Pathways [8]. In addition proteins with epitopes in the Immune Epitope Database (IEDB) [9] were included. Searches were conducted using multiple MedDRA terms for the autoimmune disease. For UniProt searches we searched the Disease Association comment field first followed by all fields. For GAD only positive associations validated by GAD curators that corresponded to MedDRA terms for the AID were used. Associations corresponding to multiple disease terms were not used, as it was often difficult to assign the association found to only one disease. For IEDB only genes coding for peptide B- or T –cell epitopes were used. To expand the list of gene associations we used Pathway Studio 9 (PS9) software [7] and searched its internal ResNet 9 Mammalian database, which contains functional relationships for humans, mouse and rat extracted via natural language processing (NLP) algorithms from the PubMed database. The PS9 gene associations were manually reviewed before adding them to gene lists from the curated databases. Review involved rapid assessments of the manuscript text for the context of a gene – disease relationship. Often this text gave a clear statement supporting, or occasionally the statement negated the association. In other cases the gene symbol/name was the same as an abbreviation for a drug or other substance in a study or the statement was otherwise ambiguous. To resolve such cases required reviewing the paper and its supplementary materials. One issue with the ResNet database was that it was not possible to determine if outcomes were derived from human, mouse or rat studies, particularly if data from human and model organisms were compared in the same manuscript. About 20% of the associations originally identified by text mining were removed after review leaving remaining 621 associations. In the process of curation a few additional genes and references were noted by curators checking the literature and added to the results.

All protein encoding genes were mapped to UniProt/SwissProt protein accessions. Functional annotations for each gene/protein were collected using UniProt APIs [13], BioMart [14] service APIs provided by Reactome and the Protein Information Resource website [15] and ID mapping services [64]. Annotations included alternate gene names, protein names, function (if known), known involvement in other diseases, pathways, and Gene Ontology (GO) [16] terms. Immune system gene classifications were downloaded from the ImmPort website (http://immport.niad.nih.gov). These annotations are included in Additional file 1. Gene networks and pathways for Influenza A and Measles infection were derived from KEGG pathways for those diseases. Only current HUGO gene symbols were used, alternate or retired gene symbols were updated via UniProt.

### Gene classifications, network, pathway and GO analysis

Genes were classified into groups using the ImmPort (http://immport.niaid.nih.gov) list of immune-related genes and were additionally classified into Immune diseases and Infectious diseases based on KEGG pathway mappings. These categories are not exclusive so a gene can be grouped in more than one. Further classification of genes related to T-cell types was done manually using the genes surface phenotype, transcription factors, secreted effector molecules and other characteristic functions as defined in (www.nature.com/nri/posters/tcellsubsets/index.html) to group the genes.

Genes associated with each autoimmune disease were used to create functional interaction networks using Cytoscape 2.8 [17] and the ReactomeFI plug-in [18,19]. The 2012 version of the FI database was used and a minimal set of linker genes were included to help connect the network. The resulting network produces a good summary of the known and predicted functional relationships derived from multiple pathway databases, protein interaction databases and the Gene Ontology [65]. Pathway and GO enrichment data were generated using the Reactome FI plug-in, excluding any linker genes from the analysis, with a P value of 0.05 or lower and false discovery rate of 0.01 unless otherwise noted. Pathway analysis for the genes in Figure 4 was done including the linker genes. Clustering the networked genes was done using a spectral partition based network clustering algorithm [66] as implemented in the ReactomeFI plug-in. Complete details of all genes and interactions plus additional images for RA, GBS, SLE and ITP are available in Additional files 5, 6, 7 and 8 respectively.

### Statistical analysis

Overlaps in genes associated with the four autoimmune diseases (Figure 1) were tested for significance in two ways. First, two-sided Fisher’s exact tests (hypergeometric tests) were performed on the overlaps for each of the 6 possible pairs of diseases. For each pair, a contingency table was constructed with the gene intersection count; the counts of genes associated with one AID and not the other, and the count of genes not associated with either disease. 9,310 disease-mapped genes (see description below of how they were obtained) were considered as the universe of discoverable genes. Second, the counts of gene overlaps were also compared to a background distribution of overlap counts. 255,970 unique disease pairs were generated, and the count of the intersection of associated genes for each disease was recorded. The gene overlaps among the four autoimmune diseases were compared to this set of values by calculating the proportion of times the AID overlap count exceeded the overlap counts in the background distribution. Overlapping pathways in the four autoimmune diseases were compared in a similar manner. For each of the 255,970 unique disease pairs, an associated set of genes was obtained for each disease in the pair. Pathway enrichment was performed on each of these sets of genes using a one-sided Fisher’s exact test, and those pathways with FDR-adjusted p-values less than 0.01 [67] were considered associated with the disease. The count of pathways in the intersection of associated pathways for the two diseases was recorded for each pair. The pathway overlap counts among the four autoimmune diseases were compared to this set of values in the same manner as the gene overlaps.

Enrichment for immune genes in each of the AIDs (Table 3) was determined using two-sided Fisher’s exact tests. The AID-associated genes were compared with the remainder of genes in the universe for each AID and immune category. P-values were adjusted using a Bonferroni correction, and only those below 0.05 deemed significant.

For these analyses the universe of discoverable genes was derived from a dataset of 34,942 unique gene–disease associations containing 9,310 unique HGNC gene names and 716 unique UMLS disease terms. The dataset was assembled from the AIDs analyzed here and additional immune related diseases we collected by similar methods and supplemented with curated disease associated genes from DisGeNet [10]. For DisGeNet associations only disease terms from curated sources were used and associations from Text-Mining sources were excluded. All diseases were mapped to common MedDRA and UMLS terminology. Detailed results from each analysis above plus additional supporting analysis and methods are provided in Additional file 9: Table S9.

## Additional files

Additional file 1: Table S1. Genes. Excel file with Genes and annotation. Additional file 2: Table S2. Sources. Excel file with source attribution for association. Additional file 3: Table S3. Vac_Ingredients. Excel file with vaccine ingredient – gene associations. Additional file 4: Table S4. Pathways. Excel file with Pathway analysis results for each AID. Additional file 5: Table S5. RA_Network. Excel file with data from RA functional interaction network. Includes Network Image, Gene Clusters and Pathway and Gene Ontology analysis. Additional file 6: Table S6. GBS_Network. Excel file with data from GBS functional interaction network. Includes Network Image, Gene Clusters and Pathway and Gene Ontology analysis. Additional file 7: Table S7. SLE_Network. Excel file with data from SLE functional interaction network. Includes Network Image, Gene Clusters and Pathway and Gene Ontology analysis. Additional file 8: Table S8. ITP_Network. Excel file with data from RA functional interaction network. Includes Network Image, Gene Clusters and Pathway and Gene Ontology analysis. Additional file 9: Table S9. Statistics.
